# Annotation, phylogenetics, and expression of the nuclear receptors in *Daphnia pulex*

**DOI:** 10.1186/1471-2164-10-500

**Published:** 2009-10-28

**Authors:** Susanne A Thomson, William S Baldwin, Ying H Wang, Gwijun Kwon, Gerald A LeBlanc

**Affiliations:** 1 Department of Environmental and Molecular Toxicology, North Carolina State University, Raleigh, North Carolina, USA; 2 Environmental Toxicology Program and Biological Sciences Department, Clemson University, Pendleton, South Carolina, USA

## Abstract

**Background:**

The nuclear receptor superfamily currently consists of seven gene subfamilies that encompass over 80 distinct receptor proteins. These transcription factors typically share a common five-domain structure with a highly conserved DNA-binding domain. Some nuclear receptors are ubiquitous among the metazoans, while others are unique to specific phylogenetic groups. Crustaceans represent the second largest group of arthropods with insects being the largest. However, relative to insects, little is known about the nuclear receptors of crustaceans. The aim of this study was to identify putative nuclear receptors from the first assembled genome of a crustacean *Daphnia pulex *http://wFleaBase.org. Nuclear receptor expression was evaluated and receptors were subjected to phylogenetic analyses to gain insight into evolution and function.

**Results:**

Twenty-five putative nuclear receptors were identified in *D. pulex *based on the presence of a conserved DNA-binding domain. All of the nuclear receptor protein sequences contain a highly homologous DNA-binding domain and a less conserved ligand-binding domain with the exception of the NR0A group. These receptors lack a ligand-binding domain. Phylogenetic analysis revealed the presence of all seven receptor subfamilies. The *D. pulex *genome contains several nuclear receptors that have vertebrate orthologs. However, several nuclear receptor members that are represented in vertebrates are absent from *D. pulex*. Notable absences include receptors of the 1C group (peroxisome proliferators-activated receptors), the 3A group (estrogen receptor), and the 3C group (androgen, progestogen, mineralcorticoid, and glucocorticoid receptors). The *D. pulex *genome also contains nuclear receptor orthologs that are present in insects and nematodes but not vertebrates, including putative nuclear receptors within the NR0A group. A novel group of receptors, designated HR97, was identified in *D. pulex *that groups with the HR96/CeNHR8/48/DAF12 clade, but forms its own sub-clade. Gene products were detected in adult female *D. pulex *for 21 of the 25 receptors.

**Conclusion:**

Nuclear receptors are ancient proteins with highly conserved DNA-binding domains. The DNA-binding domains of the nuclear receptors of *D. pulex *contain the same degree of conservation that is typically found within nuclear receptors of other species. Most of the receptors identified in *D. pulex *have orthologs within the vertebrate and invertebrate lineages examined with the exception of the novel HR97 group and the Dappu-HR10 and potentially the Dappu-HR11 receptors found in *D. pulex*. These groups of receptors may harbour functions that are intrinsic to crustacean physiology.

## Background

Nuclear receptors constitute a large superfamily of transcription regulators. They are involved in a vast array of diverse physiological functions such as the control of embryonic development, the regulation of cell differentiation, and the maintenance of homeostasis. Nuclear receptors interact with cofactors and hormone response elements (HRE) on target genes and function either as monomers, homodimers, or heterodimers [1]. Often, transcriptional regulation by nuclear receptors is dependent upon binding of receptor ligands, which typically consists of small, lipoidal molecules such as steroids and retinoids. However, for some receptors (orphan receptors) regulatory ligands are not known or may not exist [2].

**Figure 1:**
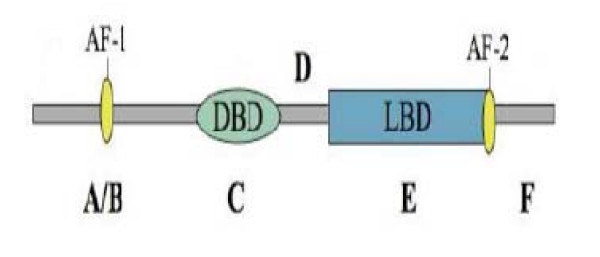
**Common structural elements of nuclear receptors**. The variable A/B region contains a transcriptional activation function (AF-1) as well as regions that are targets for post-translational modification and interact with other cofactors. The highly conserved C region or DNA-binding domain (DBD) contains two typical cysteine-rich zinc finger motifs. The D region links the DBD and the ligand-binding domain (LBD). The E region contains the LBD. This domain is involved in dimerization, contains the ligand --binding pocket, modulates positively or negatively transcription activity, and has an activation function helix (AF-2) for ligand dependent transactivation. The F region represents the COOH terminal end of the receptor.

Nuclear receptors are evolutionarily conserved proteins that are divided into seven distinct subfamilies [3] and contain a characteristic modular structure [4] (Figure 1). The A/B domains contain a transcriptional activation function (AF-1). The length of these domains is highly variable among nuclear receptors with very little evolutionary conservation. The C or DNA-binding domain (DBD) is the most conserved among the nuclear receptor domains. The DBD contains two typical cysteine-rich zinc finger motifs in tandem spanning ~80 amino acids which are directly involved in HRE recognition. The D domain functions as a hinge between the DBD and the ligand-binding domain (LBD). The LBD or E domain contains a hydrophobic ligand-binding pocket, which is specific to each receptor and is the source of the sequence variability within the LBD. In addition, the E domain mediates dimerization and ligand dependent transcriptional activation functions (AF-2) [5]. The C-terminal contains the F-domain, which is not present in all NRs and is highly diverse.

Comparative studies into functional conservation of genes and genomes will provide information on genetic diversity and similarities among major groups of organisms and give insight into protein family expansions [6,7]. Nuclear receptors have undergone many gene duplication events during metazoan evolution [8]. The estimated number of nuclear receptors is 48 in *Homo sapiens*, 21 in *Drosophila melanogaster*, and over 270 in *Caenorhabditis elegans *[9-11]. This diversity makes nuclear receptors ideal candidates for investigating the functional roles of gene duplications and phylogenetic reconstructions [12] and highlights the need to expand the repertoire of species for which nuclear receptors have been characterized.

*Daphnia pulex*, is the first crustacean for which the genome has been fully sequenced. *D. pulex *is used extensively in research studies in the fields of ecology, evolution, development, toxicology, and genetics. The recent release of the genome makes it a useful addition to the growing list of sequenced genomes for comparative evolutionary genomic research [13,14]. The aims of this study were to identify and annotate all of the nuclear receptors that are present in the *D. pulex *genome, to assess evolutionary conservation, divergence, or emergence of the crustacean receptors using phylogenetic comparison to the nuclear receptors of a vertebrate *H. sapiens*, an insect *D. melanogaster*, and a nematode *C. elegans*, and to evaluate the expression of these receptors in parthenogenetically reproducing females.

## Results and Discussion

### Nuclear receptor genes

Twenty-five putative full-length nuclear receptor genes were identified in the *D. pulex *genome. All 25 genes were identified based upon the presence of a conserved DBD. Subsequent BLAST searching of the wFleaBase database http://wFleaBase.org with each of the *H. sapiens *and *D. melanogaster *nuclear receptors, listed in Additional File 1, revealed no additional nuclear receptor sequences. Fifteen of the nuclear receptor subfamilies consist of single copy genes; two subfamilies (0A and 1H) have two paralogs each; and, two subfamilies (1L and 2E) have three paralogs each. Dappu-ECRa/Dappu-ECRb (1H), Dappu-HR97a/Dappu-HR97b (1L) and Dappu-KNR-R1/Dappu-KNR-R2 (0A) were found in close proximity as tandem repeats.

The *D. pulex *nuclear receptor family contains members that represent each of the seven subfamilies of receptors (Table 1). There are 13 members of the NR1 nuclear receptor family in *D. pulex*, which makes up 52% of the total number of nuclear receptors. In comparison, 8 out of 21 receptors (38%) of *D. melanogaster *are NR1 members. The expansion of the NR1 family is the result of the existence of the Dappu-HR10 and Dappu-HR11 receptors, an additional Dappu-EcR receptor, and the three Dappu-HR97 receptors in *D. pulex*. There are also 7 NR2 members, 2 NR5 members, and one member each of the NR3, 4, and 6 nuclear receptor subfamilies. Annotated gene and protein sequences, and *D. pulex *genome browser links for the 25 receptors are provided in Additional File 2.

**Table 1:** Nuclear receptors in *Daphnea pulex, Drosophila melanogaster, Homo sapiens *and *Caenorhabditis elegans*.

Group	*D. pulex*	Protein ID	Scaffold	*D. melanogaster*	*H. sapiens*	*C. elegans*
0A	Dappu-KNR-R1	290673	43	KNI		
	Dappu-KNR-R2	290668	43	KNRL		
				EGON		
0B					DAX1	
					SHP	
1A					THRa	
					THRb	
1B					RARa	
					RARb	
					RARg	
1C					PPARa	
					PPARb	
					PPARg	
1D	Dappu-E75	442814	1	E75	Rev-erb-a	NHR85
					Rev-erb-b	
1E	Dappu-E78	442769	23	E78		
1F	Dappu-HR3	442731	5	DHR3	RORa	NHR23
					RORb	
					RORg	
1G						CNR14
1H	Dappu-EcRa	319648	30	EcR	LXRa	
	Dappu-EcRb	442737	135		LXRb	
					FXR	
1I					VDR	
					PXR	
					CAR1	
1J	Dappu-HR96	442778	2	DHR96		DAF12
						NHR8
						NHR48
1K						NHR1
1L	Dappu-HR97a	442812	28			
	Dappu-HR97b	442724	28			
	Dappu-HR97 g	442655	40			
1 M^1^	Dappu-HR10	442777	17			
1N^1^	Dappu-HR11	316465	193			
2A	Dappu-HNF4	442738	80	HNF4	HNF4	NHR49
					HNF4G	NHR64
						NHR69
2B	Dappu-RXR	442727	328	USP	RXRa	
					RXRb	
					RXRg	
2C					TR2	
					TR4	
2D	Dappu-HR78	442757	58	DHR78		NHR41
2E	Dappu-TLL	442885	2	TLL	TLX	FAX1
	Dappu-PNR	442739	45	PNR	PNR	
	Dappu-DSF	442884	23	DSF		NHR67
				FAX-1		
2F	Dappu-SVP	442743	4	SVP	COUP-TFa	UNC55
					COUP-TFb	
					EAR2	
3A					ERa	
					ERb	
3B	Dappu-ERR	442810	11	ERR	ERRa	
					ERRb	
					ERRg	
3C					GR	
					MR	
					PR	
					AR	
4A	Dappu-HR38	442749	2	DHR38	NGFIB	CNR8
					NURR1	
					NOR1	
5A	Dappu-FTZ-F1	442811	39	FTZ-F1	SF1	NHR25
					LRH1	
5B	Dappu-HR39	442817	61	DHR39		
6A	Dappu-HR4	442822	145	DHR4	GCNF	NHR91
Total	25			21	48	284

Known receptor members for each nuclear receptor subfamily group are listed and compared to corresponding *D. pulex *nuclear receptors, including manual annotated protein identification number (Protein ID) and scaffold identity. Receptor group designations are based upon nomenclature adopted by the Nuclear Receptors Nomenclature Committee [3].

### Phylogenetic analyses

**Figure 2:**
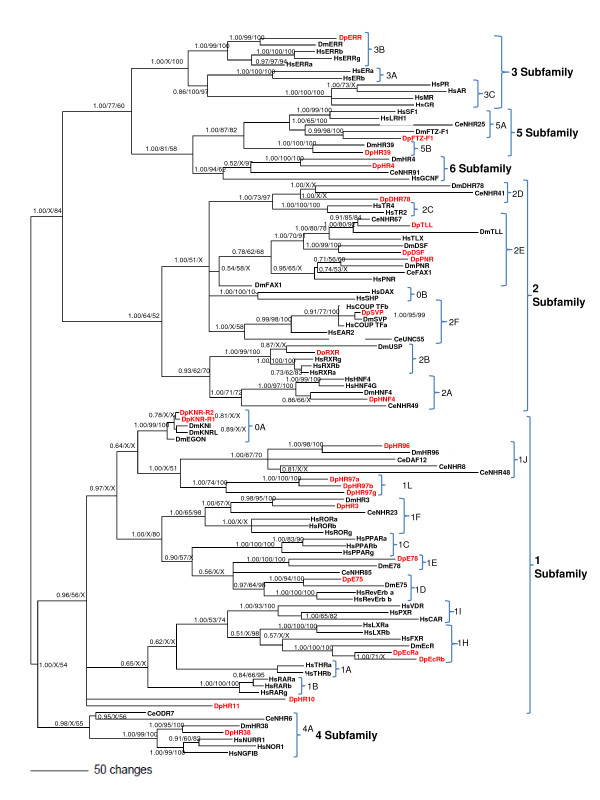
**Phylogenetic relationship of nuclear receptors in *Daphnia pulex, Drosophila melanogaster, Caenorhabditis elegans *and *Homo sapiens***. The nuclear receptors from four different species were subjected to phylogenetic comparisons using Bayesian Inference, Maximum Parsimony, and Neighbor-Joining methods. The Bayesian tree is shown with posterior probabilities from the Bayesian tree, and bootstrap support values (frequency of occurrence) from the Maximum Parsimony and Neighbor-Joining trees provided in order from left to right, respectively. Probability values are separated by forward slashes at each corresponding node; an X indicates an area of disagreement from the Bayesian tree. Notations Dp, Dm, Hp, and Ce in association with receptor names denote sequences from *D. pulex, D. melanogaster, H. sapiens*, and *C. elegans*, respectively. Numbers at nodes are posterior probabilities. All *D. pulex *sequences are in red. Figure 3 is also available in an expandable pdf format in Additional File 3

Phylogenetic analyses were performed using the amino acid sequences from the DNA and ligand binding domains and trees were constructed using three different approaches: Bayesian Inference, Maximum Parsimony, and Protein Distance (Neighbor-Joining (NJ)). Different models gave similar results at the group and subfamily levels, but Bayesian Inference showed greater resolution at the base of the phylogram. Results generated from Bayesian Inference are presented along with the bootstrap values from Maximum Parsimony and NJ (Figure 2). An expandable pdf file of the phylogram is available as Additional File 3, and a phylogram from a tree constructed from full length nuclear receptors is provided as Additional File 4. The full length phylogram showed much poorer resolution compared to the DBD/LBD-only phylogram, especially at the left hand nodes (base of the phylogram).

The *D. pulex *nuclear receptor phylogenetic tree segregates into four major clades (NR1, NR2, NR3/5/6, and NR4) each further dividing into several sub-clades. The NR3/5/6 clade further subdivides into the NR3 and NR5/6 subclades. The NR2 clade divides into two distinct subclades, and the NR1 clade divides into at least two distinct subclades. Differences between the three methods of tree construction were observed. In general, Maximum Parsimony provided the least resolution at the base of the tree.

Significant differences among the phylogenetic trees are as follows. The NJ tree placed Dappu-HR11 in the NR1A/B groups of the NR1 clade. However, no other trees including the full length trees segregated Dappu-HR11 or Dappu-HR10 into any of the NR1 groups; only as NR1 subfamily members. Dappu-HR10 and Dappu-HR11 may be sufficiently distinct from the other clade members to warrant distinct group designations (e.g., NR1M, NR1N; Table 1). The NJ tree also predicts that Dappu-EcRa is more closely related to DmEcR than to Dappu-EcRb. There is also disagreement among trees with respect to FXR's place within the NR1H group.

The Knirps that lack a LBD (group NR0A) group within the NR1 clade. It is not surprising that the NR0 subfamily members fit within other clades as the NR0 subfamily was derived to accommodate nuclear receptors that lack either the LBD or the DBD, independent of phylogenetics [3]. For example, the mammalian NR0B members that all lack a DNA binding domain, were assigned to the NR2 clade. In the Maximum Parisimony and NJ trees, the Knirps, NR1I, and NR1L groups form their own subclade, but in the Bayesian tree, these groups are separate, but within the NR1C-F groups. The exact relationship of each of the Knirps to each other is also contentious as NJ and Maximum Parsimony suggest the presence of a KNRL and an EGON in *D. pulex*, but do not agree as to the exact identity of each. Bayesian Inference suggests that the Knirps of *D. pulex *and the Knirps of *D. melanogaster *are more closely related to each other and thus duplication of the Knirps occurred after the divergence of insects and crustaceans. Analysis of the full length receptors suggests that DmEGON is orthologous to Dappu-KNR-R2 and DmKNR is orthologous to Dappu-KNR-R1. Because of the vast disagreement among methods, we named the *D. pulex *receptors Dappu-KNR-R1 and Dappu-KNR-R2 for KNR related 1 and 2.

There are few differences among the trees as they relate to the *D. pulex *NR2 receptors. For example, in the NJ tree, Dappu-HNF4 is not as closely related to DmHNF4, but instead branches from an ancestral precursor to all of the NR2A group members. Similarly DmUSP segregates from all of the RXR genes, including Dappu-RXR in the NJ and Maximum Parsimony trees. The placement of DmFAX1 and CeFAX1 is also contentious, but this was not related to any of the *D. pulex *receptors. Lastly, the placement of Dappu-HR78 differs between the three trees. The Bayesian tree shows Dappu-HR78 as members of the NR2D group. However, the NJ tree places Dappu-HR78 in the NR2C group, while DmHR78 and CeNHR41 are 2D members. The Maximum Parisimony tree has all five members related with the CeNHR41 as the most distal member. Clearly, the NR2C and 2D groups are related (Fig. 2).

There were no significant differences in the NR3, NR4, NR5, or NR6 subfamilies in the trees related to *D. pulex *receptors. The NR3 subfamily only showed one difference because the NJ tree did not agree with the other trees as to the placement of the androgen receptor. The NR4 subfamily also only showed one difference between the trees and that was related to the relationship of CeODR7 to the rest of the NR4 subfamily. All of the trees were in agreement as it related to the NR5 subfamily, and the only difference in the NR6 subfamily was due to the positioning of the CeNHR91 in the Maximum Parsimony tree.

Overall, the *D. pulex *nuclear receptor genes are distinctly placed within 18 subclades or groups based upon the phylogenetic tree (Fig. 2) and are named in agreement with the unified nomenclature system for nuclear receptors [3] (Table 1). Seven of these groups also were represented in *D. melanogaster*, *C. elegans*, and *H. sapiens *(groups 1D, 1F, 2A, 2E, 2F, 4A, 6A). The ubiquity of these groups among protostomes and deuterostomes suggests that these receptor groups evolved prior to the protostome/deuterostome divergence. Two of these groups are represented in *D. pulex and D. melanogaster *but not in *H. sapiens *(groups 1J, 2D). Representatives from these groups are also absent among the nuclear receptors of other sequenced vertebrates, including mammals and the teleost, *Fugu rubripes *[15] and may represent groups that emerged within some protostome lineage after the protostome/deuterostome divergence.

### Expression of nuclear receptor mRNAs

**Figure 3:**
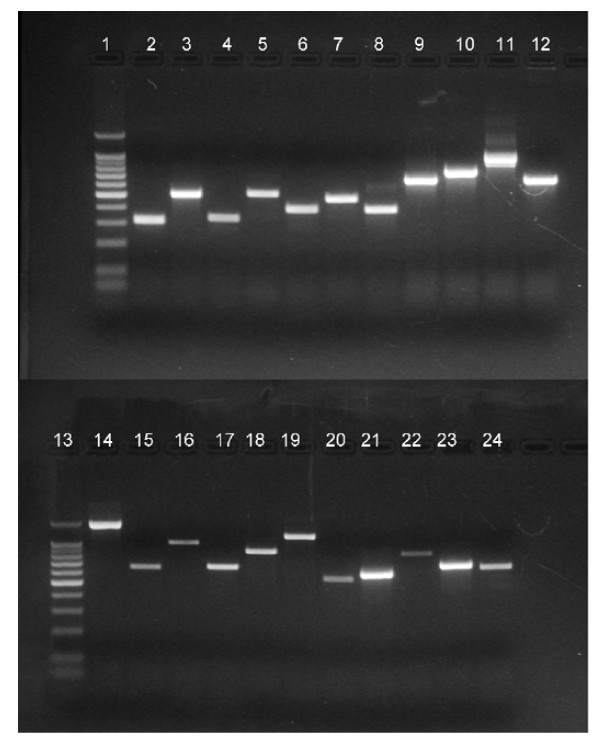
**Nuclear receptor RT-PCR products**. Products were generated using cDNA produced from total RNA extracts of adult female *D. pulex *and nuclear receptor-specific primers described in Table 2. All presented amplifications were performed as described in the Methods except for results in lanes 8-11 for which the amount of template cDNA was 2 ml with 40 amplification cycles. Gene products are as follows: **1 **and **13 **-- molecular mass ladder, **2 **-- actin, **3 **-- Dappu-HR97b, **4 **-- Dappu-SVP, **5 **-- Dappu-ERR, **6 **-- Dappu-EcR-a, **7 **-- Dappu-FTZ-F1, **8 **-- Dappu-HR3, **9 **-- Dappu-HR10, **10 **-- Dappu-KNR-R1, **11 **-- Dappu-HNR4, **12 **-- Dappu-HR11, **14 **-- Dappu-HR4, **15 **-- Dappu-HR38, **16 **-- Dappu-HR39, **17 **-- Dappu-HR78, **18 **-- Dappu-HR96, **19 **-- Dappu-E75, **20 **-- Dappu-E78, **21 **-- Dappu-RXR, **22 **-- Dappu-KNR-R2, **23 **-- Dappu-HR97 g, **24 **-- Dappu-HR97a.

Individual nuclear receptors were evaluated for the transcription of mRNA using PCR with receptor-specific primers (Table 2). RNA isolated from whole homogenates of adult female, parthenogenetically reproducing *D. pulex *yielded mRNA species that corresponded to 21 of the 25 receptors (Figure 3). All amplicons were of the molecular mass derived from the predicted mRNA sequences and nucleotide sequencing of the amplicons confirmed identity of the gene products (Additional file 5). Thus, at least 21 of the identified receptor genes are functional.

**Table 2:** Oligonucleotide primers used in RT-PCR analyses of nuclear receptor gene products.

	Primers (5'to 3')	
Gene	Reverse	Forward
Dappu-KNR-R1	CATTCGGACCTCTCCATGTT	TCCAATTCCGTCAGGATCTC
Dappu-KNR-R2	GGTTTCCATCAGCACCACTT	CTCCTCACCGACAGATCCAT
Dappu-HR11	TCACGCCAATTTCAAAAACA	GGTGGACAACGTGTCATCAG
Dappu-HR10	CACAAGCAGTGACGAGCAAT	TCCAAGCAAAAGCGAAAAGT
Dappu-E75	GGAGAACGGATCATCTTCCA	CGACAAATTCAACGGTTGTG
Dappu-E78	AGCCATTTACGACACGATCC	GGTGATGCGTTCCTGGTACT
Dappu-HR3	GAACTGTTGGCCAAGACCAT	CAAAAACAGCCGAAACCAAT
Dappu-EcRa	CATCAACCGGCTCGTCTATT	GTCGACTTTCATCTGCGACA
Dappu-EcRb	AACAATGCCGGTTGAAAAAG	TTTACCAAAGCGGAAAATGG
Dappu-HR96	CTTCGAACAAAGCGACAACA	CCTTGCAACCACCTACCACT
Dappu-HR97a	CTCGGTCCAAAACGACAACT	GTCTCCGGGTCAAATACGAG
Dappu-HR97b	TCTCAAGCTGGACAACATCG	GGAGCGTAGAAGGATTGCTG
Dappu-HR97 g	CAAGATCTGTGGGGTGTGTG	GTGTTGATTCGCCATTCCTT
Dappu-HNF4	GGATCCGCTACCAGATTCAA	TCCAGCAAGATCAACACAGC
Dappu-RXR	CGTTCAAGAGGAGAGGCAAC	GTTCGTCGGCGTAATTTGTT
Dappu-HR78	ACGTCTCGTTCCAGCTGACT	AATCCATGCGGAGGATGTAG
Dappu-TLL	GAGGAGAGCTGGAGGGAACT	GAAGTTGCTCCTCCTTGACG
Dappu-PNR	AGTATCCCAACGGAGTGACG	TGAGATGGAAGGAAGGGATG
Dappu-DSF	CTATCTGGCCCAGTGGTCAT	GCCGGAATCTTCTCTCTGTG
Dappu-SVP	CCAGGAGCAAGTGGAGAAAC	TGAAACTGGAACCGGATAGG
Dappu-ERR	TCCAACAACGTCAATCCTCA	GACTCGGACGTCACAGTTGA
Dappu-HR38	TCCGGTAGTCCACAACAACA	GGCGAAAAACTGGTGAACAT
Dappu-FTZ-F1	CTCCTACACCCCAACAGCAT	CGACAGCGACAAAAGATCAA
Dappu-HR39	AGCTGCAAGGGATTCTTCAA	CTGTTCCAGGGTGATGGACT
Dappu-HR4	TCCTCCTCCTTTGATGATGG	CGGCGGACTTTTATTGTTGT
Dappu-Actin	CCAGCCTTCATTCTTGGGTA	GGACCGGACTCATCGTACTC

We were unable to generate amplicons using various primer sets designed to amplify the products of the genes Dappu-EcRb, Dappu-TLL, Dappu-PNR, and Dappu-DSF. However, EcRb transcript is present within the EST libraries reported by the Daphnia Genomics Consortium https://dgc.cgb.indiana.edu/display/daphnia/cDNA+sequencing+project. Dappu-TLL, Dappu-PNR, and Dappu-DSF transcripts are not present in these libraries. Efforts to detect the NR2E group members are ongoing. In *D. melanogaster*, NR2E group members function in embryonic development, sexual behavior, and sex-specific neuronal development [4]. We cannot exclude the possibility that these genes are not expressed in mature, parthenogenetically reproducing females, but would be expressed during specific stages of embryo development, in sexually reproducing females, or in male daphnids.

### Nuclear Receptors of *D. pulex*

#### NR0 subfamily

The *D. pulex *genome contains two nuclear receptors that bear similarity to the *D. melanogaster *Knirp receptors KNRL and EGON (Table 1, Figure 2). Both of the *D. pulex *Knirps lack LBDs which is characteristic of the NR0A group [4]. NR0A group members were previously known to exist only in insects [16]. The ODR7 receptor of *C. elegans *is also a member of the NR0A group, based not upon its phylogenetic relatedness to other group member, but because it lacks a LBD [4]. ODR7 phylogenetically groups best with the NR4 members (Figure 2). Because direct orthology cannot be assigned, the *D. pulex *Knirps were named Dappu-KNR-R1 and Dappu-KNR-R2.

The Dappu-KNR-R1 and Dappu-KNR-R2 receptors share 62% identity to each other at the 5' end (Additional File 2). The 3' ends of the two receptors share little similarity. *D. melanogaster *KNRL possesses a 19 amino acid kni-box motif located adjacent to the zinc fingers [17]. This motif is fully conserved in Dappu-KNR-R1 and in 17 out of 19 amino acids in Dappu-KNR-R2. In *D. melanogaster*, KNRL and EGON contribute to the orchestration of embryogenesis and cell fate [17-20]. No NR0B members were identified in *D. pulex *based upon similarity to the vertebrate nuclear receptors SHP or DAX1.

### NR1 subfamily

The NR1 subfamily comprises the largest subfamily of nuclear receptors found in *D. pulex *with thirteen members. Two of the members did not associate with specific NR1 groups and were named Dappu-HR10 and Dappu-HR11 (Fig. 2); the first number marking the family designation and the second number assigned in order. Dappu-HR10 shows similarity to RAR-like receptors from lower vertebrates in BLAST comparisons. This receptor segregated into the NR1 clade during phylogenetic analyses but displayed little identity with any of the NR group members and was therefore assigned to a new NR1 group, NR1M (Table 1). Dappu-HR11 shares 60% identity with the DBD and 36% with the LBD of the sea urchin *Strongylocentrotus purpuratus *SpTHR [21]. The DBD of Dappu-HR11 shows similarity to human THR receptors in BLAST comparisons; however, little phylogenetic similarity exists between these receptors. Accordingly, Dappu-HR11 is tenuously assigned to its own group, NR1N (Table 1).

*D. pulex *possesses a single receptor of the NR1D group designated Dappu-E75 based upon its similarity to the E75 receptor of *D. melanogaster *(Figure 2). E75 previously has been identified in a decapod crustacean [22]. A NR1D member is also evident in *C. elegans *and was earlier designated as nhr-85. E75 is induced by ecdysteroids and juvenoids in insects and functions in larval molting and metamorphosis [23,24].

Insect E75 serves as a dimerization partner to HR3, an NR1F member, and in doing so, represses its action. E75 also accommodates heme and has the ability to bind small signalling molecules such as nitric oxide (NO) and carbon monoxide (CO) via this heme moiety [25]. Intracellular NO/CO binding inhibits the interaction of E75 with HR3. Thus, E75 suppresses the action of HR3 in a NO/CO permissive fashion [25]. HR3 was previously cloned from the lobster *Homarus americanus *and *D. pulex *is therefore the second crustacean species shown to possess this NR1F group member [26]. In insects [27,28] and lobsters [26], HR3 is induced by ecdysteroids and studies in insects have shown that HR3 mediates certain development-specific responses to ecdysteroid pulses. NR1F is represented by ROR in vertebrates and CHR3 (aka, NHR-23) in *C. elegans *[29].

The NR1E group was established to accommodate another rev-erb homolog initially described in insects and designated E78 (Table 1). *D. pulex *is the first non-insect that has been shown to possess this receptor gene. Like E75, insect E78 is ecdysteroid inducible and appears to contribute to the orchestration of development [30]. A function for E78 in crustaceans has not yet been established.

Analysis of the *D. pulex *genome revealed the presence of two ecdysteroid receptors, Dappu-EcRa and Dappu-EcRb, belonging to the NR1H group (Table 1). Both receptors share an overall identity of 60% at the amino acid level and 81% and 78% similarity between their DBD and their LBD, respectively. Upon activation by ecdysteroids, EcR regulates embryo development and molting in daphnids [31,32]. Interestingly, EcR has not been identified in roundworms though these organisms reportedly respond to ecdysteroid treatment [33,34]. NR1H group members in vertebrates include LXR and FXR.

The daphnid genome contained one nuclear receptor with significant homology to insect HR96. This receptor was assigned to the NR1J group and was designated Dappu-HR96. This represents the first identification of a NR1J group member in a crustacean. Dappu-HR96 contains the conserved 13 sequential amino acid residues and the base-contact residues ESCKAFFR, that are unique among all NR1J group members [35]. The NR1J group appears to be unique to ecdysozoans but the DBD shares similarity with the DBD of the vertebrate VDR (NR1I). Previous phylogenetic analysis has indicated that the the NR1I and NR1J groups share a common receptor ancestor but diverged between the Deuterostomes (NR1I) and the Protostomes (NR1J) [36]. Little is known of the function of HR96 receptors in ecdysozoans. However, recent studies revealed that DHR96 might play a role in xenobiotic stress responses in *D. melanogaster *[37]. Additionally, DAF-12, a NR1J member in *C. elegans *has been implicated in diapause [38].

Receptor gene identification and accompanying phylogenetic analyses revealed that *D. pulex *possesses a unique group of nuclear receptors consisting of three genes that share sequence similarities with HR96 and to a lesser extent VDR. These receptors have been tentatively provided the group designation NR1L and the receptor names Dappu-HR97a, Dappu-HR97b, and Dappu-HR97g (Table 1, Figure 2). Dappu-HR97a and Dappu-HR97b are tandem duplicated genes with a DBD and LBD that are 100% and 78% identical, respectively. Phylogenetic analyses (Figure 2) suggest that the ancestral Dappu-HR97a/b was derived from the duplication of the HR97g gene. Both Dappu-HR97a and Dappu-HR97b lack a conserved intron found within Dappu-HR97g, DHR96, and Dappu-HR96. The GT/AG sites that are required to form this intron are absent from Dappu-HR97b, but not Dappu-HR97a. However, this potential intron is not excised from the expressed Dappu-HR97a mRNA (Additional File 2 contains the genome browser links to the Dappu-HR97 genes). At the amino acid level, Dappu-HR97a and Dappu-HR97g are 41% identical, with a 63% and 45% identity between their DBD and LBD respectively. Dappu-HR97g contains the same conserved base-contact residues (ESCKAFFR) found in all NR1J members (see above) while Dappu-HR97a and Dappu-HR97b have one amino acid difference. The DBD/LBD domains and full-length proteins of each of the Dappu-HR97 receptors cluster within the HR96/DAF12/NR0A group; however, Dappu-HR97 comprises its own subclade resulting in its unique group designation.

The function of the Dappu-HR97s is not known. The similarity of its LBDs to HR96 and the NR1I subfamily suggests a role as a xenobiotic or endobiotic sensor. In contrast, the similarity of the DBD to the NR0A members (Knirps) suggests a role in development. It is interesting to speculate that the Dappu-HR97s may play a role in mediating developmental and life-stage changes such as male offspring and diapause egg production that are mediated by environmental cues.

### NR2 subfamily

The *D. pulex *genome contains a single member of the NR2A group that has been designated Dappu-HNF4 due to its similarity to HNF4 in *H. sapiens *and *D. melanogaster *(Table 1). An HNF4 ortholog in *C. elegans *is known as NHR49. Dappu-HNF4 is the first NR2A member to be described in a crustacean. Dappu-HNF4 possesses the P-box sequence DGCKG, which is characteristic of members in this group [16]. Expression patterning and gene deletion experiments in *D. melanogaster *indicate that HNF4 is involved in orchestrating the development of components of the digestive system [27,39].

RXR, a NR2B group member, is represented in the *D. pulex *genome and has been designated Dappu-RXR (Table 1). This receptor has been cloned previously from crustaceans including *D. magna *[40]. RXR is ubiquitous among the Bilateria with the notable exception of *C. elegans *[36]. This gene is best recognized in insects as the partner to EcR. Retinoids, most notably 9-cis-retinoic acid, serve as a high affinity ligand to RXR in vertebrates [41] and molluscs [42]. Terpenes such as juvenile hormone and methyl farnesoate have been proposed as ligands to insect and crustacean RXR [40,43,44].

The NR2C and NR2D groups represent different groupings within related subclades (Figure 2). The *D. pulex *genome contains one member of this clade that we have designated Dappu-HR78. Dappu-HR78 shows greater phylogenetic relation to the NR2D members from *D. melanogaster *(DHR78) and *C. elegans *(NHR41), than to the NR2C members from *H. sapiens*, TR2 and TR4. Numerous NR2C/D orthologs have been identified in a variety of species. In vertebrates, TR2 and TR4 have diverse functions and are able to either activate or repress target genes [45]. Recent studies suggest that TR2/4 might play a role in sequential, gene-autonomous silencing of the e and g -globin genes during development [46]. The function of HR78 in arthropods is still unknown but it has been shown to be induced by 20-hydroxyecdysone and to inhibit the transcriptional activity of EcR in a reporter gene assay [27,47]. Dappu-HR78 represents the first NR2C/D member identified in a crustacean.

The *D. pulex *genome possesses three receptor genes from the NR2E group (Table 1). Dappu-TLL is a homolog to TLL of *D. melanogaster *and TLX of *H. sapiens*. Dappu-PNR is a homolog to PNR of insects and vertebrates. Dappu-DSF is a homolog to DSF in *D. melanogaster *(Table 1). The NR2E group members are important in aspects of neural and optic development in both insects and vertebrates and appear to be ubiquitous among the Bilateria [34]. Two receptors, FAX-1 and CeNHR67 are found in *C. elegans *[48]. The daphnid NR2E members possess the unique structural features of other NR2E genes including a very short A/B domain and a characteristic P box sequence (CDGCSG, CDGCAG, or CNGCSG) [49-51]. A NR2F group member that is homologous to SVP in *D. melanogaster *and COUP-TF in vertebrates is represented in the *D. pulex *genome as Dappu-SVP (Table 1). Dappu-SVP possesses two DBDs which is characteristic of NR2F members based upon the conserved domain database (CDD) [52]. This is the first report of a NR2F receptor gene in a crustacean. COUP-TF in vertebrates and SVP in insects have been shown to be potent transcriptional suppressors that negatively regulate the transcriptional activity of other nuclear receptors such as EcR, RAR, VDR, TR, and HNF4 [53-55].

### NR3 subfamily

The NR3B group is represented by a single gene in the *D. pulex *genome designated Dappu-ERR (Table 1). Estrogen-related receptors (ERRs) are ubiquitous among metazoans including *Trichoplax *[56] a basal metazoan [57]. Yet, aside from known importance in placental development, little is known about its function [58,59]. Recent studies suggest that ERR could be part of the estrogen signalling and metabolism pathway [60].

### NR4 subfamily

A single gene in the *D*. pulex genome represents the NR4A group and is designated Dappu-HR38 (Table 1). This group is ubiquitous among the Bilateria and homologs include NGFIB, NURR1, and NOR-1 in vertebrates; HR38 in insects; NHR6 in *C. elegans *(Figure 2), and NR4A5 in *Schistoma mansoni *[34,47,61,62]. In *D. melanogaster*, DHR38 plays an important role in cuticle formation and is induced by ecdysteroids [63]. HR38 may be a true orphan receptor since its 3D structure revealed no ligand-binding pocket and coactivator-binding site. These unique features seem to be conserved in vertebrate NGFIB members and might indicate similar biological functionalities [64].

### NR5 subfamily

*D. pulex *contains two genes in the NR5 subfamily, designated Dappu-FTZ-F1 (NR5A) and Dappu-HR39 (NR5B) (Table 1). Dappu-FTZ-F1 is highly conserved among the Bilateria [65]. In *D. melanogaster*, FTZ-F1 orchestrates stage-specific responses to ecdysteroids [27,30]. Two NR5A nuclear receptors are present in mammals, SF1 and LRH1. SF1 is a transcription factor for genes involved in reproductive physiology and endocrine functions [66,67], while LRH1 regulates genes important for cholesterol metabolism [68]. Dappu-FTZ-F1 possesses a sequence that is highly conserved in all NR5A members called the FTZ-F1 box (Table 3) which is located immediately after the DBD and plays an important role in the high affinity interactions of the receptor with DNA [69].

**Table 3:** Amino acid sequence of the FTZ-F1 box of various NR5A group members.

Gene	FTZ-F1 box
Dappu-FTZ-F1	AVRADRMRGGRNKFGPMYKRDRARKLQVMR
DmFTZ-F1	AVRADRMRGGRNKFGPMYKRDRARKLQVMR
HsSF1	AVRADRMRGGRNKFG**F**MYKRDRA**L**K**Q**Q**KKA**
HsLRH1	AVRADRMRGGRNKFG**F**MYKRDRA**L**K**Q**Q**KKA**
CeNHR25	AVRADRMRGGRNKFG**SF**YK**K**DRA**HRM**Q**RNA**

Bolded amino acids indicate differences from Dappu-FTZ-F1. Amino acid sequences were derived from [82].

NR5B previously was known to exist only in insects as HR39. The revelation that this group exists in crustaceans suggests that this group emerged in arthropod evolution prior to the divergence of insects and crustaceans. The function of the NR5B gene product in arthropods is unknown. Sequence similarities between FTZ-F1 and HR39 suggest that NR5B in arthropods is the result of a duplication and subsequent divergence of the NR5A receptor [70].

### NR6 subfamily

The *D. pulex *genome contains a nuclear receptor gene bearing significant homology to the NR6A members DHR4 in *D. melanogaster*, GCNF in *H. sapiens*, and NHR91 in *C. elegans*. This is the first report of an NR6A group member in a crustacean. Expression patterns of DHR4 in *D. melanogaster*, suggest that this receptor is regulated by ecdysteroids and may play a role in the molting process [27,30]. DHR4 can act as a repressor or an inducer while coordinating growth and maturation in *D. melanogaster *[71]. In vertebrates GCNF contributes to the regulation of embryonic development, reproduction, and neurogenesis [72].

## Conclusion

Thus far, 25 nuclear receptor genes have been identified in *D. pulex*. Eighteen receptors are present as a single copy gene in the *D. pulex *genome. One receptor (Dappu-HR97) contains three paralogs based upon phylogenetic analysis, of which two are in tandem repeat. Two receptor genes are present with two paralogs (Dappu-KNR-R1/R2, Dappu-EcRa/b). All of the nuclear receptor groups that are present in *D. melanogaster *are also represented in *D. pulex*. This observation indicates that these receptor groups emerged prior to the divergence of the Insecta and Crustacea. (Insects and crustaceans diverged over 500 myr ago [73]). This conclusion is substantiated by the presence of these receptor groups in either of the non-arthropod species *H. sapiens *or *C. elegans *(Table 1). At least two novel receptor groups were discovered in the *D. pulex *genome designated NR1L (receptors Dappu-HR97a, Dappu-HR97b, and Dappu-HR97g), NR1M (Dappu-HR10), and potentially NR1N (Dappu-HR11), which may be have orthologs in the sea urchin and also shows significant similarity to NR1A/B group members using some distance methods. The NR1L and NR1M groups were not represented in any of the other genomes used in the phylogenetic comparisons and may represent receptor groups that emerged among the crustaceans subsequent to divergence from the insects.

While *D. pulex *possesses two members of the NR0A group, KNR-R1 and KNR-R2, *D. melanogaster *contains three distinct members, KNRL, KNI and EGON. The absence of KNI in *D. pulex *might indicate that this NR0A group member is a product of gene duplication that occurred in insects after the divergence of insects and crustaceans or was lost in *D. pulex *and potentially other crustaceans.

Nuclear receptors are ancient proteins that have ubiquitous important functions in regulating many aspects of metazoan physiology. Biological functions have been established and ligands identified for some of the receptors. However, the function of many of these family members remains elusive. Daphnids have a long history of use as a model organism in studies of ecology, toxicology, and evolutionary biology. The elucidation of the *D. pulex *genome creates an invaluable resource to study the functional role of specific genes in the well-characterized physiology of this organism. The identification of the nuclear receptor genes in *D. pulex *was the first step to gain more insight into the evolutionary and structural aspects of these important transcription factors. The foundation is now established to link specific nuclear receptors with physiological function.

## Methods

### Identification of nuclear receptors in *D. pulex*

Analysis with the Basic Local Alignment Search Tool (BLAST) [74] was performed using the highly conserved DNA-binding domain sequence of *Daphnia magna *RXR [40] against assembled *D. pulex *transcripts from the JGI Genome Portal http://www.jgi.doe.gov/Daphnia. All sequences containing a DNA-binding domain were aligned in Vector NTI (Invitrogen), assembled into larger contigs, and analyzed for redundancy. Open reading frames were determined by translating all DNA sequences into protein sequences as described below. A BLAST search was conducted with the fully assembled transcripts against the NCBI database for further identification and against the newly constructed wFleaBase database http://wFleaBase.org to identify each putative nuclear receptor (Table 1). In addition all *H. sapiens *and *D. melanogaster *receptors were used in a BLAST search against the FleaBase database in an effort to identify any nuclear receptors in *D. pulex *that escaped detection in the initial search (Additional File 1). All nuclear receptor protein sequences were run through the NCBI program Simple Modular Architecture Research Tool (SMART) for the identification of the DBD and LBD [75].

The nuclear receptors were manually curated to identify open reading frames and protein sequences (Additional File 2) with the help of filtered gene models developed by The Joint Genome Institute (JGI) http://www.jgi.doe.gov/Daphnia/ and wFleaBase http://wfleabase.org. Manual curations were based on the v1.1 gene builds, comparisons to other genomes such as human, mouse, *Drosophila*, zebrafish (*Danio rerio*), Xenopus, and bovine (*Bos taurus*) available on the *Daphnia *genome portal, knowledge of intron-exon borders in related genes, BLAST searches, and DNA sequences of PCR products [76,77].

Nomenclature for the nuclear receptors of *D. pulex *was based on the sequence similarity and phylogenetic analysis of the full length sequence to that of nuclear receptors present in *H. sapiens and D. melanogaster*. The name of the closest ortholog either from vertebrate or insect was chosen for *D. pulex*, except for Dappu-HR10 and Dappu-HR11, which were named based on their location within the NR1 family. Based on phylogenetic analysis, Dappu-HR97a, Dappu-HR97b, and Dappu-HR97g were found to comprise a distinct group designated NR1L. The letters a, b, or g after each HR97 receptor name refers to the Greek letters alpha, beta, and gamma.

### Sequence alignments and phylogenetic analysis

The DNA and ligand binding domains of each receptor were isolated using the conserved domain database (CDD) that is part of BLASTP [52]. The DBDs and LBDs of each receptor were combined and then aligned. Receptors from *D. pulex *were compared to nuclear receptors from human (*H. sapiens*), fruit fly (*D. melanogaster*), and nematode (*C. elegans*) using publicly available sequences from GenBank. The amino acid sequences of each *D. pulex *receptor used in the phylogenetic analysis is available in Additional File 2, and the NCBI accession numbers of each nuclear receptor used in our analyses are available in Additional File 1. To construct phylogenetic trees, all of the *D. pulex*, *H. sapiens*, *D. melanogaster*, and *C. elegans *amino acid sequences were aligned using default parameters in ClustalX [78]. Trees were constructed using Bayesian Inference, a probabilistic model-based method of phylogeny reconstruction that is similar to maximum likelihood but which has substantially reduced computation time. Bayesian trees were constructed with the software MrBayes version 3.1.2 [79] on the freely available computing cluster Bioportal http://www.bioportal.uio.no run by the University of Oslo. Phylogenetic trees were constructed using the "mixed-model" approach in which the Markov chain Monte Carlo sampler explores nine different fixed-rate amino acid substitution models implemented in MrBayes. We used 4 chains with runs of 5 million generations with chains sampled every 100 generations and with a burnin of 5000 trees; the *WAG *[80] model was selected as the best fitting substitution model by MrBayes. Phylogenies were midpoint rooted in which the root is placed halfway between the two most divergent sequences due to the difficulty in choosing an outgroup for such a diverse and ancient gene family and we have successfully used this approach previously [76].

Maximum parsimony and neighbor-joining (NJ) distance parameters were used to provide additional phylogenetic support for classification, naming, and the phylogenetic relationships observed between *D. pulex *and the other specie's nuclear receptors. Unrooted parsimony was constructed using PAUP version 4.0b10 [81] with heuristic searches, tree-bisection-reconnection, topological constraints not enfoced, and multiple tree option in effect with an initial maximum tree setting at 10,000. Branch support was measured by bootstrapping with 500 replicates. Distance parameters were also measured using PAUP 4.0b10 by NJ with default characteristics (mean character difference and among site rate variation). Branch support was measured by bootstrap analysis with 1000 replicates.

### PCR amplification of *D. pulex *nuclear receptors

Total RNA was extracted from 40 adult female *D. pulex *using the SV Total RNA Isolation System (Promega). The isolated total RNA was converted to cDNA with the Promega ImProm-II Reverse Transcription System using oligo (dT) primers. One μl of undiluted cDNA was used as template for PCR amplification using the GoTAQ system (Promega) under the following conditions: 94°C-two minutes, thirty cycles at 94°C-30 seconds, 55°C- one minute, 72°C- one minute, final extension at 72°C for ten minutes. All primers were designed with Primer3 software and were based on the predicted cDNA sequences from FleaBase. The sequences of all primers used in this study are described in Table 2. Amplification products were isolated by electrophoresis using a 2% agarose gel, visualized with ethidium bromide, excised from the gels, and recovered using the WizardTM purification system (Promega). Nucleotide sequences of the amplification products were determined (SeqWright Inc., Houston, TX) (Additional File 5).

## Authors' contributions

SAT carried out the sequence identification, contributed to the phylogenetic data analysis, and contributed to the preparation of the manuscript. WSB manually annotated the receptors, contributed to phylogenetic analysis and manuscript preparation. YHW assisted in the comparative analyses of nuclear receptors from different species. GW performed all PCR analyses. GAL coordinated the study and was responsible for the final drafting of the manuscript. All authors read and approved the manuscript.

## Supplementary Material

Additional file 1 **Sequence identification of nuclear receptors**. NCBI accession numbers of all *D. melanogaster, H. sapiens*, and *C. elegans *nuclear receptors used for the alignments and phylogenetic analysis are listed. Click here for file

Additional file 2 **Detailed summary of each NR sequence, including its position in the *D. pulex *Dappu v1.1 draft genome assembly, annotated open reading frames and corresponding amino acid sequences**. An Excel workbook (xls) table of each of the NR genes and found in the *Daphnia pulex *genome, their nucleotide and amino acid sequences, links to their scaffold position, manual protein identifying number, and presence of an expressed sequence tag (EST) within the "chosen one". Click here for file

Additional file 3 **Expandable pdf file of the nuclear receptor phylogenetic tree provided as Figure **2. Phylogenetic relationship of nuclear receptors in *D. pulex, D. melanogaster*, *C. elegans*, and *H. sapiens*. The nuclear receptors from four different species were subjected to phylogenetic comparisons using Bayesian Inference, Maximum Parsimony, and Neighbor-Joining. The Bayesian tree is shown with posterior probabilities from the Bayesian tree, and bootstrap support values (frequency of occurrence) from the Maximum Parsimony and Neighbour-Joining trees provided in order from left to right, respectively. The probability values are separated by forward slashes at each corresponding node; an X indicates an area of disagreement from the Bayesian tree. Notations Dp, Dm, Hp, and Ce in association with receptor names denote sequences from *D. pulex, D. melanogaster, H. sapiens*, and *C. elegans*, respectively. Click here for file

Additional file 4 **Phylogenetic tree of full length nuclear receptors**. Phylogenetic relationship of full length nuclear receptors in *D. pulex, D. melanogaster*, *C. elegans*, and *H. sapiens*. The nuclear receptors from four different species were subjected to phylogenetic comparisons using Bayesian Inference, Maximum Parsimony, and Neighbor-Joining. The Bayesian tree is shown with posterior probabilities from the Bayesian tree, and bootstrap support values (frequency of occurrence) from the Maximum Parsimony and Neighbor-Joining (Distance) trees provided in order from left to right, respectively. The probability values are separated by forward slashes at each corresponding node; an X indicates an area of disagreement from the Bayesian tree. The non-Bayesian trees were not able to distinguish separate subfamilies. The phylogenetic comparisons were done as described for the LBD/DBD trees, except the burnin was 10,000 for the full length Bayesian Inference analysis. Notations Dp, Dm, Hp, and Ce in association with receptor names denote sequences from *D. pulex, D. melanogaster, H. sapiens*, and *C. elegans*, respectively. Click here for file

Additional file 5 **Nucleotide sequences of nuclear receptor RT-PCR products**. Nucleotide sequences of RT-PCR products depicted in Figure 3. The sequences confirmed expression and contributed to the manual annotation of the nuclear receptors. Result present the consensus sequence of both DNA forward and reverse strands unless indicated otherwise with 'forward' or 'reverse' indicating the strand sequenced. Click here for file
