# Supplementary material for: Annotation, phylogenetics, and expression of the nuclear receptors in Daphnia pulex
Source: BMC Genomics. 2009 Oct 28;10:500. doi: 10.1186/1471-2164-10-500 (PMC2774871; doi:10.1186/1471-2164-10-500)
Supplement: Additional file 1 — Sequence identification of nuclear receptors. NCBI accession numbers of all D. melanogaster, H. sapiens, and C. elegans nuclear receptors used for the alignments and phylogenetic analysis are listed. [file 1471-2164-10-500-S1.doc]

**Additional File 2:** Sequence identification of all nuclear receptors from this study. NCBI accession numbers are shown for the nuclear receptors in *D. melanogaster*, *H. Sapien*, and *C. elegans*. For *D. pulex*, the JGI genome portal version 1.1 protein identification numbers are given for each nuclear receptor.

| **Group** | *D. pulex* | Protein ID | *D. melanogaster* | Accession Number | *H. sapiens* | Accession Number | *C. elegans* | Accession Number |
| --- | --- | --- | --- | --- | --- | --- | --- | --- |
| **0A** | DpKNR-R1  DpKNR-R2 | 290673  290668 | KNI  KNRL  EGON | CAA31709  AAF51627  CAA34626 |  |  |  | AAC46497 |
| **0B** |  |  |  |  | DAX1  SHP | AAH11564  AAH30207 |  |  |
| **1A** |  |  |  |  | THRa  THRb | AAH08851  AAI06931 |  |  |
| **1B** |  |  |  |  | RARa  RARb  RARg | AAH08727  AAH60794  AAA63254 |  | AAC46497 |
| **1C** |  |  |  |  | PPARa  PPARb  PPARg | AAB32649  AAA36469  AAH06811 |  |  |
| **1D** | DpE75 | 442814 | E75 | AAN11687 | Rev-erb-a  Rev-erb-b | AAH56148  AAH45613 | NHR-85 | AAO39185 |
| **1E** | DpE78 | 442769 | E78 | AAF51692 |  |  |  |  |
| **1F** | DpHR3 | 442731 | DHR3 | AAA28461 | RORa  RORb  RORg | AAH08831  AAH93774  AAA64751 | NHR-23 | P41828 |
| **1G** |  |  |  |  |  |  | CNR14 | AAA96982 |
| **1H** | DpEcRa  DpEcRb | 319648  442737 | EcR | AAF52702 | LXRa  LXRb  FXR | AAV38218  NP_009052  AAI30574 |  |  |
| **1I** |  |  |  |  | VDR  PXR  CAR | AAB95155  AAD05436  AAY56401 |  |  |
| **1J** | DpHR96 | 442778 | DHR96 | AAC46928 |  |  | DAF-12  NHR-8  NHR-48 | AAD34462  AAP31437  CAD36502 |
| **1K** |  |  |  |  |  |  | NHR-1 | AAC48174 |
| **1L** | DpHR97a  DpHR97b  DpHR97g | 442812  442724  442655 |  |  |  |  |  |  |
| **1M** | DpHR10 | 442777 |  |  |  |  |  |  |
| **1N** | DpHR11 | 316465 |  |  |  |  |  |  |
| **2A** | DpHNF4 | 442738 | HNF4 | AAF52702 | HNF4  HNF4G | NP_849180  NP_004124 | NHR-49 | CAD57702 |
| **2B** | DpRXR | 442727 | USP | AAF45707 | RXRa  RXRb  RXRg | AAI10999  AAC18599  AAA80681 |  |  |
| **2C** |  |  |  |  | TR2  TR4 | AAA36761  NP_003289 |  |  |
| **2D** | DpHR78 | 442757 | DHR78 | Q24142 |  |  | NHR-41 | NP_500073 |
| **2E** | DpTLL  DpPNR  DpDSF | 442885  442739  442884 | TLL  PNR  DSF  FAX-1 | AAB71371  AAF58145  AAF52303  AAF54133 | TLX  PNR | AAL05871  AAD28301 | FAX-1  NHR-67 | AAD55066  CAA97428 |
| **2F** | DpSVP | 442743 | SVP |  | COUP-TFa  COUP-TFb  EAR2 | AAH04154  AAH42897  AAH02669 | UNC55 | CAP16277 |
| **3A** |  |  |  |  | ERa  ERb | AAI28575  AAV31779 |  |  |
| **3B** | DpERR | 442810 | ERR | AAL37554 | ERRa  ERRb  ERRg | AAH63795  AAC99409  EAW93335 |  |  |
| **3C** |  |  |  |  | GR  MR  PR  AR | AAH15610  AAA59571  AAA60081  EAX05380 |  |  |
| **4A** | DpHR38 | 442749 | DHR38 | AAF53914 | NGFIB  NURR1  NOR1 | CAG32985  AAH09288  CAI95138 | NHR-6 | CAA85271 |
| **5A** | DpFTZ-F1 | 442811 | FTZ-F1 | AAN11667 | SF1  LRH1 | AAH32501  AAI18572 | NHR25 | CAA91028 |
| **5B** | DpHR39 | 442817 | DHR39 | AAN11107 |  |  |  |  |
| **6A** | DpHR4 | 442822 | DHR4 | AAX73355 | GCNF | AAB96828 | NHR91 | CAB60329 |
