# Supplementary figures and images for: Annotation, phylogenetics, and expression of the nuclear receptors in Daphnia pulex
Source: BMC Genomics. 2009 Oct 28;10:500. doi: 10.1186/1471-2164-10-500 (PMC2774871; doi:10.1186/1471-2164-10-500)

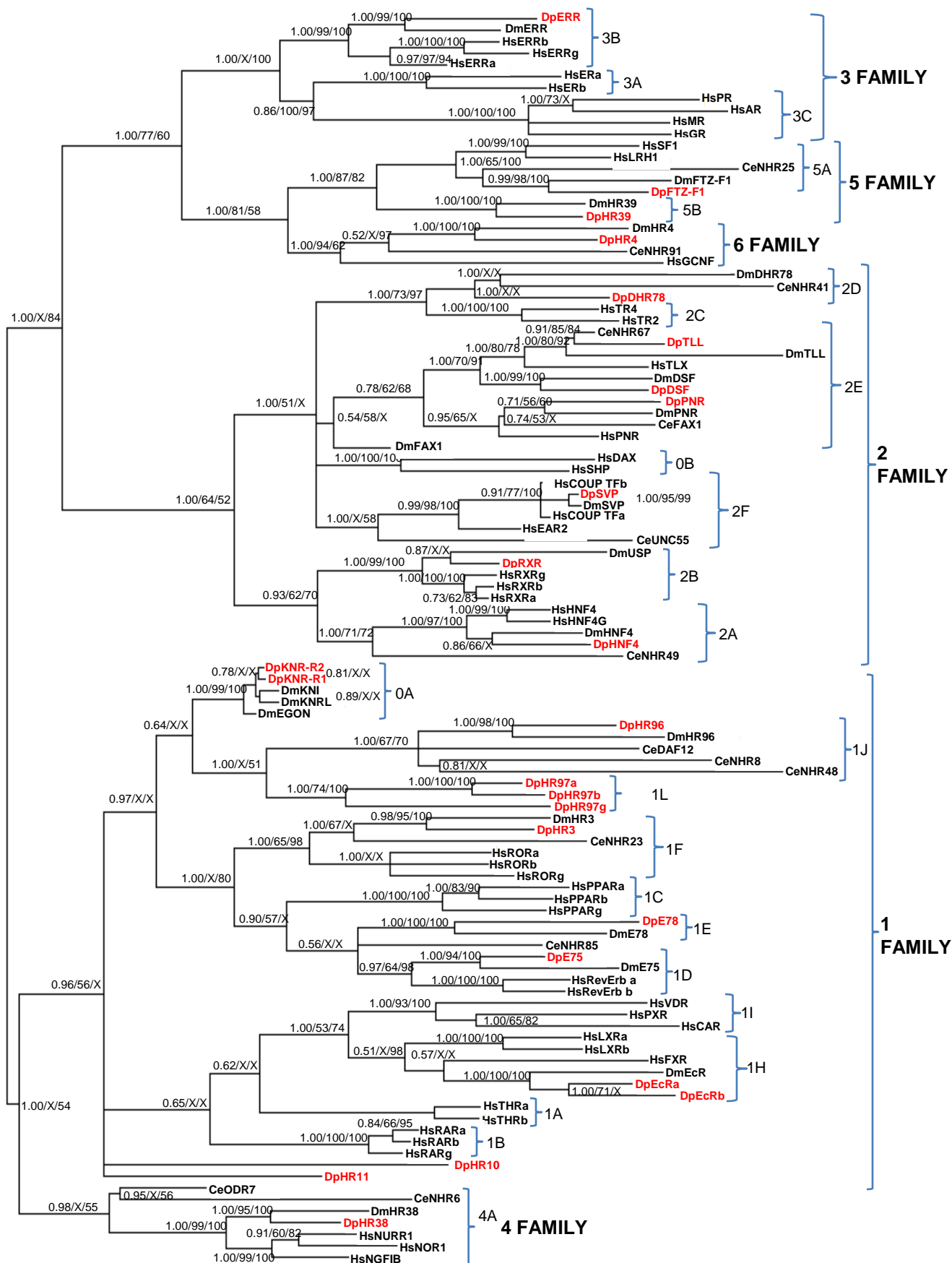

50 changes

Supplement: Additional file 3 — Expandable pdf file of the nuclear receptor phylogenetic tree provided as Figure 2. Phylogenetic relationship of nuclear receptors in D. pulex, D. melanogaster, C. elegans, and H. sapiens. The nuclear receptors from four different species were subjected to phylogenetic comparisons using Bayesian Inference, Maximum Parsimony, and Neighbor-Joining. The Bayesian tree is shown with posterior probabilities from the Bayesian tree, and bootstrap support values (frequency of occurrence) from the Maximum Parsimony and Neighbour-Joining trees provided in order from left to right, respectively. The probability values are separated by forward slashes at each corresponding node; an X indicates an area of disagreement from the Bayesian tree. Notations Dp, Dm, Hp, and Ce in association with receptor names denote sequences from D. pulex, D. melanogaster, H. sapiens, and C. elegans, respectively. [file 1471-2164-10-500-S3.pdf]
