# Supplementary material for: Annotation, phylogenetics, and expression of the nuclear receptors in Daphnia pulex
Source: BMC Genomics. 2009 Oct 28;10:500. doi: 10.1186/1471-2164-10-500 (PMC2774871; doi:10.1186/1471-2164-10-500)
Supplement: Additional file 5 — Nucleotide sequences of nuclear receptor RT-PCR products. Nucleotide sequences of RT-PCR products depicted in Figure 3. The sequences confirmed expression and contributed to the manual annotation of the nuclear receptors. Result present the consensus sequence of both DNA forward and reverse strands unless indicated otherwise with 'forward' or 'reverse' indicating the strand sequenced. [file 1471-2164-10-500-S5.doc]

**DpActin**

TTCTTGGGTATGGAGTCTTGCGGCATCCACGAGACCACCTACAACTCGATCATGAAGTGCGACGTCGACATCCGTAAGGATCTGTACGCCAACACTGTCCTGTCTGGTGGCACCACCATGTACCCAGGCATTGCTGACCGCATGCAGAAGGAAATCACCGCCTTGGCTCCATCCACCATGAAGATCAAGATCATTGCTCCCCCCGAGCGCAAGTATTCCGTCTGGATCGGTGGCTCCATCTTGGCCTCTCTGTCCACCTTCCAACAGATGTGGATTTCCAAACAAGAGTACGATGAGGTCCGGTCCA

**DpKNRL**

TCATTTGGGNACCTCTCCATGTTCAAACATCATCACGCCCTGGCCACCAGTAGCAGAAGCCCCGGACGGATGCAGCAATTCCGACACGGCCGGCGACAAGGAGAAATCAGGTCACAACAACAACAACAACAACAACAACATGTCCTCCTCCTCGTCGGTGGCATCCGGGCGCAGTGATTCGCCCATGGATGACATCAACGACGACTGCGCCGCATCCAAGGAAAATCTGCGGATGCTTTTGACTGGAAGTCCGTCCGAGTCGTTCAACATTTTCCACCACCACTACCACAACCACATGCGGAAGAGTTCCAGTTCCACGTCGCCCATCAGAGGAGGAGGAGGGGGACTCTTGGCCATTGCCGGAATGCCTTTCGGTCACCTCCACCACTCGACTGCGTCATCGCCGCTTGTCGATCCCGTCCGGACGAGTAGCCCCAAACTGCCGGGCGAATCCAGCGGCGGATCGGCTAGCGGACATCGATCTCTGGTTCACCCGAGTAGTCATCTCCTTTACCCGCTGATCGCCCGCCACCACCAGGCCGCCCTGCATCCGTACCACCGGTCACCGCCGACTCACGACATGCGGAGGTCGAGAAGTCCGGCCTCCGCCGTCCCCAGTCCGGTGGATTCTGACGTTCCGGAACAAGACGGTCCCATCGATTTGTCCTGCAAGGCTTCGTCCTCTTCGTCGTCGTCGTCGCCATTCGGCCACCAGCAATCCATGTGCCACATCCGGGCGGAGATCCTGAGG

**DpEGON**

CATTCAGCACCACTTGATGACCGCGGCCGCCATGGTCGTGGGTGGCCCCGGAGTACCGTCCAGCATGTCCAGCAACGAGTCGAACAAAGATGACGCCGAAGCCGAGTGCAAGAATTCGTCGGCCTCTCCGTCCGTCAGCTCGCCCGGATCACCTCACCACTCGGATTCTTCGCTGGAGTTGGATCCGTCTTCGGTGCCCAGCGGGGAGCGAAGCAGTGGCAAGCAGAGCCACCGAGGGCACCACAACAACCACCACAGCAGTTTACATCACCACGCCGCACTGGCCAGTCTGGCCGGTCACCACCACAATCCTTTCGGTCTGAAACAGCCGTTCAATCCGGCCGATTTCTTCCGGCCTTTGGGTATGCCGATGCTTCCGCCTCACTTGCAAGCAGCGGCGGCGGCTGCGGCGGCTGCTGCGGCTGCGGCCAACCTCCACCATCACCACGGATTCTATCCGCCAAACCTTCCGGCGCCTAGGCTCCTGTTCCCAGCCGGAATGGGCTTCCCGTCGTCGGTCAACACGCCGCCCACCAGTCTCAGCGGCAGCGGGTCCAAGTCTCACAGCTCCTCCTCGGCCACGCCCAACTCACTTCTGCTGGATGCCGCCGGAGCGAGAGCCGCCAAAAAGTTCAACATCGAGTCGATGCTTCATCTCCACCAGCAGCAGCAACAGCAGCAACAGGAACAGATGCAGAAACAACAACAACAGCAACAACTCAAGCGTTCTACCTCGTCATCGTCGCTCAAGCTGACGCCCCCGCCGTCTCAGCAGCGATCGGCGCCGGCGGAGGAGGAGCAGGACACGCCGATGGATCTGTCGGGTGAGGAGAAA

**DpTHRL**

CCAAATTCAAAAACAACCGTCGTCAAGAGACGTTTCCCCCAACAGGTGATCCAGCGCAATCCCGTGAACGAGCTACTTGGTCCAATCGTTCGTGCTTATCGGGAGACTTTCACTAGAGTCGGTCCCAGTCGGAGTAATTCCCTGCTTCACGTAGACGACACCAATTCTATGGGAGTTCTTCAGATTGGTGACCTTTTAACGCCAACTATTACCAAAGTCATCACGTTTGCTAAAGCCCTTCCTGGGTTCAACACTTTCTCTACAGACGACCAGATTTGCCTATTAGGAGGATCGTGTATGGAGGTTCTCTGTCTGAGAGTAGCACTGAGATTTGACGTTGAACGTCAAGGATTCCATTTGCGAGGTGAACGTTTTCTGACTCGAGCCGATATTTTAGCTCGACCGGAGCTGGGAATGTTGTCGCCACTTTTTAACTACGCACAACGCGTAGCTGATTTGAAATTGGACGAAGCCGAGGCCGCCACTGTGATGGCTACGTGGATTCTTCAATCATCTAGACCTGATTTGAAAGAACCAGAAGGTGTAGAGGTGTTCCAGGAATCAATTATGAACGTTTCCATGTCGTACGCCAAGAATGGAGCTCACGCTTCGGACGAACTGCGGTGGCTGAAAACGCTGATGACACGTTG

**DpRARL**

CAGTGACGAGCAATATGGCAGTTACCACATCGAGATGCCACCATGTAGTGTTTGTGGTGACATCAGTTGCGGCATTCATTACGGAGTTGTGGTTTGTGAAGGCTGCAAGGGCTTTTTCCGACGAGCGAATTTAAGAACCCCAGAAGAGCCGGCGTTTATATGCTTTTACAGTTCAAACTGTATCGTCAATCGTTCAAATCGAAATAAATGTCGAGCTTGTCGATTGCGTCGGTGCTACGAAGTTGGAATGGTTTTTGGTGATGGATGCGAGTTGAGCAAAAAGCGCCGTCGCAATCGATATGGACTGTCNCAGGATCCACCTCTCCCACCACCGATTCATTGTCGGCCGCGGTCACTGCCATCGCCGGACGTACTAGAATGGATGGGTTTNGTTGTAGCAGCTCAATCGGCAAGTTTTACTNCCGTTTCTGAAATATTCGCTTCAAGGTATGCTGCCGTCTCGTCATTAGGTCCGGATCAACTGTGGCCACCTTGCGGGGATCTCAGTCATCACGGTTATCAGGCTATCCAACATTTTGCCCATTCTCTACCGTTTATCGCTTTCTTGCCTCCTGATTTGCGAGGCTTACCCGGTAGAAACGAATGCACTTGATGTGATGATTTTGCGACTAACTTTTCGCTTTTGC

**DpE75 (forward)**

CTGATGAGGATGGTCGAGTCGCCTCCGCGGACCACAGTGCTGGAGCCGGAAGTAATGCTGGCAGCAGCTGCCCTTATTCAAAGATGCGCAAATTGGATTCTCCGGATGATTCCGGAATCGAATCGGGTGTGGATCGCTACGAGAAAATGTCGACGGCTTCCCGGTCGACCAACACGTCCCTTTGCTCATCACCGCGCTCTTCGCTGGAAGACAAAGTCAAGGAAGTGGATGAGATGCAGCAACATCATCAACACCACCATCATCATCATCAT

**DpE75 (reverse)**

GATTCGGCTGTTGCTGTTGTTGTTGTTGTTGCGGAATTCGGCTGCTGCTGGGACTTGGGCTAGAGTGGTGATGTGGAGGAGACACTGGCGACGACCTCTGATGGTGGTGGTAATTCCAGCTAGATGGCGAGGATGAGCCGGCGTAAAGTGGACCGGAAGTCAAAACGGTTCTTTGTTGTTGTTGTCCGTTACTGCTGCTGCTGCTGGAAAGGGGTGACGGACAATAGTTTTGGCTGGACGATGAAGGTGTGGGCGATGAGCCCGTCACGTTGCCCTTCATGATGTTGGCGACGATGAGAT

CGGCGCGGCGCTGCTGTTCGGTGCTGACCATTTTGGGACCTTCCATCAGGGAACGGGCCAGGGTCGAGTGGGTCGACGTCAGCGAACTGACGACGCCCGAAGACAACGAAGACGCCAGCAACGAAGACGACGACGTCGACGGAGGTGACGTCAAGGCACTGCACAGGGCAGAGTACGTGCCCGGAGGGGAACTGAGAGCCGCAGACAAGACGCTCACGGTCGACGTAGAC

**DpE78**

CGACACGATCCTAACCGTGTCGCAAGCCCATCACGCTCATTGTGCCTACACAGAAGAGAAGACCCGCAACATTGTCCGCACTCCCGTACCCATCGTGATGGACGAAGGTGGGAACAACGACAANGCGGATGAAACCCGACGAATAGCCACCTGGTCGGCGCTGGCCACTCACATGACGCCGTCCATCCAGCGCGTCGTCGAATTCGCCAAACGTGTTCCAGGTTTCCCGGAGTTGAGTCAGGACGATCAGTTGATCCTGATCAAGATTGGATTTTTCGAAGTGTGGCTGGGCCACGTCAGTCGACTGATCAACAGCCAGGAAGGAACGATGACATTGACGGACGGTGTGACCCTGTCCAAGCAACAGATGGACCTCATCTTTGACGCTGATTTCATCCAGACCGTCATGGGATTCGCCGAAGGCTTGAACCAAATGTCGATGAACGATAGTGAAATGGCTCTTTTTTCGGCCGCCGTTCTCCTATCNCCCGATCGGCCCGTCATTAACGACGCCAAGGGCATCGCTCAGTACCAGGAACGC

**DpHR3**

GAACTGTTGGCCAAGACCATCGGCGACGCTCACAGCCGGACGTGTCTCTTCAGCGGGGAGCACATCGCCGACATGTTGCGCAAACCGCAGGATATTTCTAAAGTGCATTATTACAAAAACATGGCACAAGAGGAGCTCTGGTTAGAGTGCGCCCAAAGATTGACGGCTGTTATTCAGCAAATTATCGAATTTGCCAAAATGGTTCCCGGATTCATGAAACTTTCTCAGGATGATCAGATTGTTCTTCTGAAAACAGGGAGCTTTGAGCTGGCCGTTTTGCGGATGAGTCGCTACTACGACCTGAGCCAGAACGCGGTGCTGTTCGGCGACACGCTCCCTGCCGGTCGAGGCTTTTCTAACGCCGGATTCAGTTGAGGCCAAATTGGTTTCGGCCTGTTTTTGAA

**DpEcRa**

TCATCAACCGGCTCGTCTATTTCCAGGAAGAATTCGATCAACCGTCCGAAGAGGATCTGAGAAAAATTTCCACGTCGGGCATTCACGAATCGGACGCCGACGCCAAATTCAAACACATAACGGAGATGACGATCCTGACTGTGCAGCTGACGGTCGAGTTTTCCAAGCGGCTGCCGGGCTTCGACACGCTCCTGCGCGAGGATCAAATCACGTTGCTCAAGGCCTGCTCGAGCGAGGTGATGATGCTGCGCTGCGCCCGACGCTACGACGCCAACACGGACTCGATCGTCTTCGCCAACAACCTGCCCTACACGCGCGAGTCGTACAACATGGCCGGAGTCGGAGACACGGCCGAGCCGCTCTTCCGCTTCGGCAAGAGCATGTCGCAGATGAAAAGTCGACAA

**DpHR96**

CAGAGGAAGAATGCTTGAAATGTCTCCTCCGGAGGATTGCCCAAGTGACAGCTCTCCGTCTACCGCCAATGNNGGGATGCGTAGTCAATCCATGAGCGTAGCCGATGCCTTTAATATAAACTTGCAGACCATGGATGCTTTATTAAATACTGCCATTTCAGCTGAATACAATGTCGTGGTCGATTTAGTTCGGGGCAATGATACTAGCCCACTGTCGCCAGTCCGTGATCGCCAACTGAATGAACTGGAAATGGCCAAGTTGCAGGAACTCGTGGTGGCAAACAAAGCCCTTTTGGCACCCTTGACGGAAGAACGGCCAGTCGATCTTCATTACGATTCGGGGGACCCAACATTACTAAATGTTATCAATTTGACGGATATNGCCATCAGGCGAATCATCAAAATGGCGAAGAAATTNGCAGCTTTCAAGACTTTGTGTCAAGAGGACCAGATCGCCTTGCTGAAGGGTGGCTGTACCGAGCTGATGATTTTGCGCTCGGTCATGTCCTACGACGCCGACAAAGGATGCTGGAAGATACCACACACGGACTCGCACATGAATCACATCAAAGTGGAGGTGTTGAAGGAAGCCCAGGGAAACTTGTACGAGGAACACCAACGCTACATTCAATCTTTTGATCCGCTGTGGCGTTCAGACGAGCACATCATGCTGCTGCTGTCGGCCATCACTTTATTCGATCCCAACCGACCTCACGTCATCCATCGAGATGCCATCAAATCTGGAACAAGAGTCGTACTACTATTTGCTTAGGCGCTATTTGGAGTCAGTGGTAG

**DpHR97a**

CCGGGTCAAATACGAGGGCTCCACGCAGCAGACACATCTCCAGGACGCCACCTTTCAGCAGGCAGCTCTGATCCTGGAAAGGTAAACTCGAAAAGTCGGCCATCAGTCGGGAGAAAAACGCAAATCGCCGAATCAGGGTCCCAAACATCTCCATAACCTGGACACCGGGACGGTCGCTATCGAAGAAACGCAGTTCTGAGCGATAGGGAACGTGTTGGTAGGCGTGCATGTATTTGGTGACGATCGATTCGATTTCTTTGATTTCGAGCGGAGCCATAAAATCGATCATTCGTTCGATCTGAGGCTCGTAGTCAGCACTGGCAACGGCAGCGCTGGTGGGGGCAGTAGGAGCAGCGAATCCAGGACTCTGATCTCCTCCGCTCGAGTTGGACGAAGTATTTAAATTTTGAGCACTCGCATTGGAGCTAGTTGACACGGTAGGCGACCTTTTGCACGATTTCTTCTCGGCCCTGGCCTTCATGAGAGCCTTGCGCTCCTCTTCGGTCATGACCCAGCTCTTCTCCATGCCGATGGCGAAGCATCGCTTCATGCGGCAGTACTGGCAACTCCTTGCGGTTCGACAGGGAAATGACCGCACTGGCCGCGGTGACAGCACTGGAAGTGGAGGTAGTTGTCGTT

**DpHR97g**

TGTGGGGTGTGTGGTGATAAGGCAAAAAGTTACCACTTTGGTGGACTCTGTTGTGAATCCTGCAAAGCCTTTTTTCGAAGAGCAATGCAAAATGATTCCTTCAANGCATTTTTCTGTGTCCACGGCCAATCTTGTATTGTAAACAAGGAAAATAGAAGATCTTGCCAGAAGTGTAGGATCCAGAAATGTTTCTCAATAGGAATGGAGAAAGGATGGCTCCGAACTGATGAAGAAAATGTGAAAATGAGAGAAGCGAAGCTAGTCAATAAGCCTGCAAAGCAAAATGCTGAGTTAGATGAGCAGCGAAGCAGCAGTCCTGATTCAGCTTCGCAAAACACTTCTCCCTACCAACCCAGATTTGAAAATATCCTTGTTTATCTGATGCCAGAGGATGTGAAATTTTTAGAAAAAATTGTCGATGACTATTGGAAAGCTTACAANCAAGTACCTTTTGGAGCCGAAGTGACGCAGAACAGTACACCACGAAGCGGTTTGCAAGTGATNAGCATGTTCACCACAGGCATCCGCCGATGGGCTGCCTTTGCCCGCTCTCTGACCGAGTTCTCTTCTCTCTGCCGACAAGATCAAGCGACCCTATTGCA

**DpHNF4**

CAACATTTGAAGGAGCTTCACTTGAATCGGTTGGTAATGCAAACAATTCTGCCCCTTCGTCGGGGTACAATCCGCCCGTTGTGGTCCCATCTACACTGACCGCAATGGCTCCACTTTCAGCAGATTCGTCTCCCACGCACTCAACGGGTCCATCTCTTACAACTCTTCCTCTCCTGAATGGGCAAAACGGATTAGGTGGGCATCAGTCCATGATGATGGACCGTGGACCACCGCTGACCATGTCTCCAGACTCTGAGGATGACGCCTTTCCCTTGGGAGCATTGTCCAATCACCACCACCAGCCTTTCAAACAAGAGGTGACGGAAGGAATGGACAATGCGTACACATAGATACTTGACCGTTCCCGAATTTTACAAAAAGAAAGAAAAATGATAAAAAAAGAGAAAAAAAGGGTTTTGTTTTGTTAAGTTAAAGTTTTAAGAGATAATCTATTCGATTTGAGAATTGCGCGGCAAATATTTTCCAAAGATGAGTAGCACAATTACTATAACAGGGACTCAACATATCCATCGTGTTGCTACCATCAGGGTTTTTTTTTCCATTCAAATGTGCGTCATTTCTATTAAAAACAAACGAGTTAATTGCTTCTGATTGACCTGTGTCGAACTTCATAGTTGCTCAATATAATTGTTAATTAGATAGGGGCCNAAATACATCTGACAAACTGGTAATTGAATCTTAAACAGTCACTTCTGCAAATTTTTCTCTTTCTGGGACTGGCTA

**DpRXR (reverse)** TGCGAAACAGATTTTAAGCCCTTGGGATCTGGGTTGAACAAGATGATGGCTCTCAGACAACCCAATTCGGCCAGGTCGAGTTTCATTTCCCTCATTTTGGAGACGAGTTCGGTAAGCACACGATCAAAGATCGATCCGACGCCAGCCTGGTGAGCCGAGTTCCTGTGAATAACAAGTCCAGTAGCAAGGACGATCCCGTCCTTGACACCGACCGAACGATGAGAAAATGCAGCAATCAGCAGTTCATTCCAGCCAGCTCTGAGCAAAACGACTTGGTCGTCAAGGGGCAATTCCGTGAAA

TGTGGGATGTGTTTAGCCCACTCAACCAGTTGGAAAAGTTGCTTATCTGTAGCAGCGCATATATTGCCTAGAGCGGCAGTGGCTGAATTCACCTGGGGCTCGTCCTTGCACTCGACACGTTTTTCAGCTTCCAAAACTCGATCGATTGGCATGTCACCTTGTCCACC

**DpHR78**

ACGTCTCGTTCCAGCTGACTACGCCGTCACCTATGCCCGTCTTCCTCAATGCTCATTTCATCTGCGAGAGTGCTTCGCGGCTCCTGTTCCTCTCGGTACACTGGGCACGATCCATCGGCGCCTTCCAGCTGCTCTCGTCCGACACTCAAATCGAACTTGTTCGCGGCTGCTGGTCCGAACTCTTTGCCCTGGGCATGGCCCAGTGCTCTCACATCATGTCCCTGCCGGCCATTCTGACGGCCATCATCACCCACCTTCAAGCCTCCGTCGCCCAGGACAAAGTCTCTGCACAGCGAGTCAAATTGGTGACGGAGCACGTTTTGCAGTTGCAAGATTACGTCAACACCATGTCCAAGCTACAGGTCGACGAGCACGAATACGCTTACTTGAAAGCCATTGCCCTCTTTAGTCCAGATCACGCGGGGTCGACTGGCCGTCAGGTGGAGCGTTTCCAGGACAAGGCTGTGAAAGAATTGCGGACGTACGTGACGCAAACGTGGAACGAAGAGGCTGAGGACCGATTCCCACGGCTCCTGTTGCGGCTTCCGCCGCTCCGCTCACTCCAGCCGGGATCTGATGGAAGAGCTCTTTTTCGCCGCTCTGATCGGCACCGTCCACATCGACTCGGTCATTCCCTACATCCTCCGCATGGATT

**DpSVP**

TTCCAGGAGCAAGTGGAGAAACTCAAAGCCCTCCACGTCGACTCGGCCGAATATTCTTGTCTCAAAGCCATCGTCCTATTCACCACAGACGCTTGCGGATTGTCGGACGTGGGACACATCGAGAGCCTGCAGGAGAAATCGCAGTGCGCCCTGGAAGAGTACTGCCGGACGCAGTACGCCAACCAACCGGTCCGTTTTGGCAAACTGCTCCTGAGGCTGCCTTCTTTACGTACCGTCTCGTCGCAAGTCATCGAGCAACTCTTCTTCGTCCGGCTGGTCGGCAAAACGCCCATCGAGACACTCATCCGCGACATGCTCCTATCCGGTTCCAGTTTCAAA

**DpERR**

AACGTCAATCCTCAACCCGTCAAAAAAGTCTCGCTGGAAGATAACAAAATCTTGAACACGCTGGGACAGTGTGAACCCGAAATGTTGACGACGGTCGACATTTTGTGGCCGGAAGGCGATGCTTCGAGTATGGATCCATCGTTGCGGATCCTTTGCACATTGAGCGAACTCTACGATCGGGAATTGGTGGCCGCCATTGGTTGGGCTAAACAGATTCCAGGGTTTCTGGAAATGCCCCTCAACGACCAGATGAGGCTGCTGCAGACGAGCTGGCCGGAGGTACTGACCCTGTCGCTGGCCTTCCGCTCCATCCCGTTGAACAGTAGCAATCCGAAATTGCAATGGTCGGCTGACTTTGGCATGAACGAGAAGGAAGCACGCGAATGCGGCATGGAAGAACTCTTCTTCCAGTGCGTGCAAATAGCCCAGCGGCTGGANCAGCTGTCGGTGACCCGTGAGGAGTACTATCTCCTGAAGGCCCTCGTGCTGGTCAACTGTGACGTCCGAGTCA

**DpHR38 (forward)** AGCTTCGAGCGATCCGTTCCGGCCGTAGACAAGTGCAGCATCAGTCAAGGCGTTTCGGCTGGACGATCTGATTCATCGGCGTCTCGATCCAAATCTCCGTCGCAATTGTGCGCCGTTTGCGGCGACAATGCCGCCTGTCAACACTACGGCGTCCGCACTTGCGAAGGCTGCAAAGGATTCTTCAAGCGAACAGTACAAAAAGGCTCCAAGTATGTCTGCCTGGCCGATCGGAGTTGTCCGGTCGACAAGAGACGTCGCAATCGCTGCCAGTTCTGTCGCTTCCAGAAATGCCTCTCTGTC

GGCATGGTCAAAGAGGTCGTGAGAACAGATTCTTTGAAAGGACGACGAGGAAGATTGCCATCGAAACCGAAATGTTCGCAAGAGGCATCC

**DpFTZ-F1**

TTCTCCTACCACCCCAACAGCATTCCTCCACGTCATCCGTCTCGCCCAAAACCTTCCATTTCGAGGGACTTTTGACCGGTAACACCATGACTGGGCAACAACAACGGTCCCAATGTAGGAGCTGGATCGGTTAGCGGAGCCAAAGTTCCACCATTGATCCGCGAATTCGTCCAGTCTCTCGACGACAAAGAGTGGCAGAGTGCCCTGTTTGGTCTTTTACAGAGTCAAACGTACAATCAGTGTGAAGTGGATTTGTTTGAGCTGCTCTGCAAGGTGCTGGACCAGAACCTTTTTACNCAAGTGGACTGGGCACGTAATTCCTACTATTTCAAGGATCTAAAGGTTGACGATCAAATGAAATTGCTGCAACACGCTTGGTCGGACTTGTTGATTTTGGATCATTTACATCAAGCGATTGCACCAATCCACCTGCCTGACGAATCTTCGCTGCCCAATGGTCAAAAGTTTGATCTTTTGTCGCTGTCGA

**DpHR39 (forward)** GGCGCCGCCTGTCCCGTCCCATCGCCACACGCAAAAAGTGTCCGGCCTGTCGATTTGAAAAGTGCCTCAACACCGGCATGAAACTGGAAGCCATCCGCGAGGATCGAACTCGAGGCGGACGGAGCACTTACCAGTGTACTTATGCCGTGCCCAGCGGGTCGGGAGCGTCGACGGGTCTTGTGGGCGGCGATCCGTCGGTGGGCGGTTCTTCCTCGACTGGAAACGGAGGCGGAAGCAGCGGAGGCAAAGTCCTGCTGACGCCCAACGGCGACAGCAGCAGCTCCAGGTCGATGGATTCATCCGAGTGGAGCTCGTCGCCTCCGTCGGCCACTCCGGCCGGAGGTGGTGGTGCCCATTCTCATCACCATCACCACGACCACGGCCA

**DpHR39 (reverse)** GCCGCCGATCCGTCCGACTCGTTGCAGTGCCACAAATGCTCCACGTTCATAATTTCGAGGAGGAGCTGCGGAACGGCTGCCGACGAGTTGCTTGGATGGTGCGGATGCTGGTGGTGCAGCAACAGGTGGTGGTTGTTGCTGTGGTGGTTGTTGTGATGGCCGTGGCCGTGGTGGTGGTGATGATGATG

**DpHR4 (reverse)**

ACAGCGCCGGAATCCTTCCGCCGGGCATCCTGTCCTCTCTGACGGCGGCCAGGACCATGCCCTGGGCGAGGCACTTTTGAAAGCGGCAATACTGGCACCGATTCCGCTGGGCCTTTGTGATCTCGCAATTGCCTTCCGCCACGCAAGTGTACACCCGCTTATTCTGCACCGTCCGCTTGAAAAAGCCTTTGCACCCTTCACAGGTGATGATTCCGTAATGGAGTCCGGTGGCTTTGTCCTCGCAGATCATGCAAATCATGGGCGTGTCTTCATCGTCCTCCTCCATCCGGTTGCCGTCGCCGTGGCTGTTGCTGCCGTTGACGGCGGACGGGCGACCTCCGCCGTTGATGGTCGTCACGATGGACGACGGAAGCTGATGGATGGCCGATGTCGGGTGATTATGAGTGCCGTTGTCATGGTGACCGTTGCTGTGAAGGTGATTGCTGTTGTTGTGGTGGACATTGTTGGAGCTGACGATGATGGACGACCGACCGGAACAGACGGAATAATCCAAAGCCTGCGAATCCGGCCTCTGCTGCTGCTGCTGCTGTTGTTG
