# Supplementary material for: Annotation, phylogenetics, and expression of the nuclear receptors in Daphnia pulex
Source: BMC Genomics. 2009 Oct 28;10:500. doi: 10.1186/1471-2164-10-500 (PMC2774871; doi:10.1186/1471-2164-10-500)
Supplement: Additional file 4 — Phylogenetic tree of full length nuclear receptors. Phylogenetic relationship of full length nuclear receptors in D. pulex, D. melanogaster, C. elegans, and H. sapiens. The nuclear receptors from four different species were subjected to phylogenetic comparisons using Bayesian Inference, Maximum Parsimony, and Neighbor-Joining. The Bayesian tree is shown with posterior probabilities from the Bayesian tree, and bootstrap support values (frequency of occurrence) from the Maximum Parsimony and Neighbor-Joining (Distance) trees provided in order from left to right, respectively. The probability values are separated by forward slashes at each corresponding node; an X indicates an area of disagreement from the Bayesian tree. The non-Bayesian trees were not able to distinguish separate subfamilies. The phylogenetic comparisons were done as described for the LBD/DBD trees, except the burnin was 10,000 for the full length Bayesian Inference analysis. Notations Dp, Dm, Hp, and Ce in association with receptor names denote sequences from D. pulex, D. melanogaster, H. sapiens, and C. elegans, respectively. [file 1471-2164-10-500-S4.pdf]

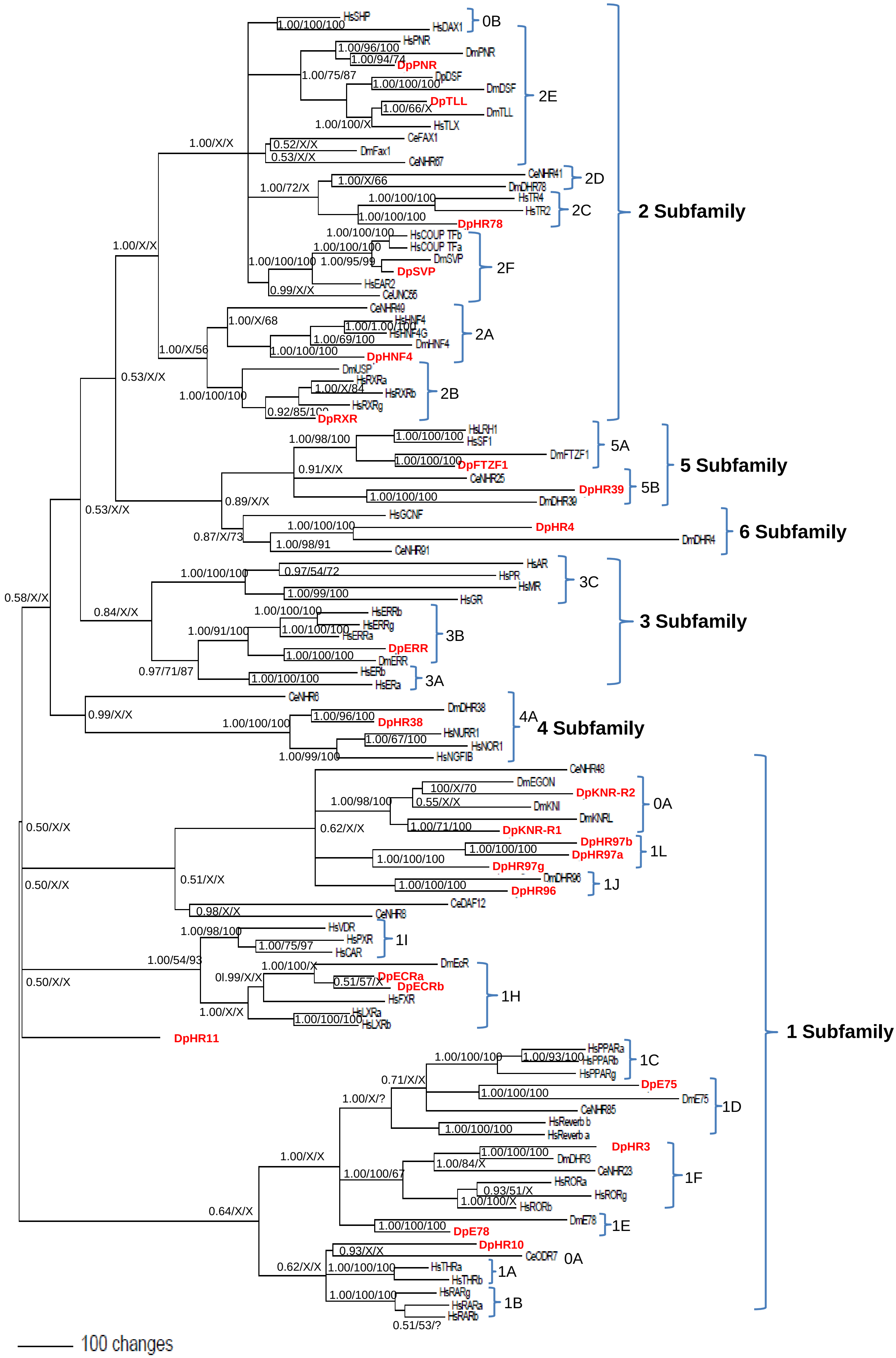

HsSHP
1.00/100/100
HsDAX1
0B
HsPNR
1.00/96/100
DmPNR
1.00/94/74
DpPNR
1.00/75/87
DpDSF
1.00/100/100
DmDSF
DpTLL
1.00/66/X
DmTLL
2E
1.00/100/X
HsTLX
1.00/X/X
CeFAX1
0.52/X/X
DmFax1
0.53/X/X
CeNHR67
CeNHR41
1.00/X/66
2D
DmDHR78
1.00/72/X
1.00/100/100
HsTR4
HsTR2
2C
1.00/100/100
DpHR78
2 Subfamily
1.00/100/100
HsCOUP TFb
1.00/X/X
1.00/100/100
HsCOUP TFa
1.00/100/100
1.00/95/99
DmSVP
DpSVP
2F
HsEAR2
0.99/X/X
CeUNC55
CeNHR49
1.00/X/68
HsHNF4
1.00/1.00/100
HsHNF4G
2A
1.00/69/100
DmHNF4
1.00/X/56
1.00/100/100
DpHNF4
0.53/X/X
DmUSP
HsRXRa
1.00/X/84
HsRXRb
2B
1.00/100/100
HsRXRg
0.92/85/100
DpRXR
HsLRH1
1.00/98/100
1.00/100/100
HsSF1
5A
DmFTZF1
1.00/100/100
DpFTZF1
0.91/X/X
5 Subfamily
CeNHR25
DpHR39
5B
1.00/100/100
DmDHR39
0.89/X/X
0.53/X/X
HsGCNF
1.00/100/100
DpHR4
6 Subfamily
0.87/X/73
DmDHR4
1.00/98/91
CeNHR91
HsAR
1.00/100/100
0.97/54/72
HsPR
3C
HsMR
1.00/99/100
HsGR
0.58/X/X
1.00/100/100
HsERRb
3 Subfamily
0.84/X/X
HsERRg
1.00/100/100
1.00/91/100
HsERRa
3B
DpERR
1.00/100/100
DmERR
0.97/71/87
HsERb
1.00/100/100
3A
HsERa
CeNHR6
DmDHR38
0.99/X/X
1.00/96/100
DpHR38
4A
4 Subfamily
1.00/100/100
HsNURR1
1.00/67/100
HsNOR1
1.00/99/100
HsNGFIB
CeNHR48
DmEGON
100/X/70
DpKNR-R2
1.00/98/100
0.55/X/X
DmKNI
0A
DmKNRL
0.50/X/X
0.62/X/X
1.00/71/100
DpKNR-R1
DpHR97b
1.00/100/100
DpHR97a
1L
1.00/100/100
DpHR97g
0.50/X/X
0.51/X/X
DmDHR96
1.00/100/100
1J
DpHR96
CeDAF12
0.98/X/X
CeNHR8
HsVDR
1.00/98/100
HsPXR
1I
1.00/75/97
HsCAR
1.00/54/93
1.00/100/X
DmEcR
0I.99/X/X
DpECRa
0.51/57/X
0.50/X/X
DpECRb
HsFXR
1H
1.00/X/X
HsLXRa
1.00/100/100
HsLXRb
DpHR11
1 Subfamily
HsPPARa
1.00/100/100
1.00/93/100
HsPPARb
1C
HsPPARg
0.71/X/X
DpE75
1.00/100/100
1.00/X/?
DmE75
1D
CeNHR85
HsReverb b
1.00/100/100
HsReverb a
DpHR3
1.00/X/X
1.00/100/100
1.00/84/X
DmDHR3
CeNHR23
1F
1.00/100/67
HsRORa
0.93/51/X
HsRORg
1.00/100/X
HsRORb
0.64/X/X
DmE78
1.00/100/100
1E
DpE78
DpHR10
0.93/X/X
CeODR7
0A
0.62/X/X
1.00/100/100
HsTHRa
1A
HsTHRb
HsRARg
1.00/100/100
HsRARa
1B
HsRARb
0.51/53/?
100 changes
